# Poor mood after oral contraceptive use is associated with increased vulnerability to peripartum depression, premenstrual dysphoric disorder, and higher genetic risk for depression

**DOI:** 10.1007/s00737-026-01708-z

**Published:** 2026-05-06

**Authors:** Jacqueline Kiewa, Penelope A. Lind, Ian B. Hickie, Sarah E. Medland, Brittany E. Mitchell, Christel M. Middeldorp, Nicholas G. Martin, Naomi R. Wray, Enda M. Byrne

**Affiliations:** 1 Institute for Molecular Bioscience, University of Queensland, Brisbane, Australia; 2 QIMR Berghofer Medical Research Institute, Brisbane, Australia; 3 School of Biomedical Sciences, Queensland University of Technology, Brisbane, Australia; 4 Brain and Mind Centre, The University of Sydney, Sydney, Australia; 5 Child Health Research Centre, University of Queensland, Brisbane, Australia; 6 Public Health Research Institute, Amsterdam University Medical Centers, Amsterdam, Netherlands; 7 Department of Psychiatry, University of Oxford, Oxford, UK

**Keywords:** Oral Contraceptive Pill, Mood effects, Depression, Reproductive depression, Genetics

## Abstract

**Purpose:**

This study tested whether adverse mood effect of the oral contraceptive pill (OCP) is associated with reproductive depressive episodes, including peripartum depression (PPD), premenstrual dysphoric disorder (PMDD), and perimenopausal depression.

**Method:**

In a sample of 3,547 OCP users from the Australian Genetics of Depression Study, who reported a lifetime depression diagnosis, logistic regression was used to test the association of PPD, PMDD, and perimenopausal depression with OCP adverse mood effect. Polygenic scores (PGS) for major depression (MD) were also tested for association with adverse mood effect. Sensitivity analyses tested for modification of these associations by a history of depression prior to first OCP use (prior depression), or by depression onset before the age of twenty (child/teen depression onset).

**Results:**

Adverse mood effect was reported by 1,342 OCP users (38%). PPD, PMDD, prior depression and child/teen depression onset were significantly associated with adverse mood effect (PPD: Relative Risk (RR) = 1.66,CI=[1.4–2.0], *P* = 2.0 × 10^− 6^; PMDD: RR = 3.78,CI=[2.4-6.0], *P* = 2.2 × 10^− 8^; prior depression: RR = 1.32,CI=[1.1–1.5], *P* = 5.9 × 10^− 4^; child/teen depression onset: RR = 1.56,CI=[1.3–1.8], *P* = 1.1 × 10^− 7^). The association of PPD with adverse mood effect remained significant for women with no prior or child/teen depression onset (RR = 1.77,CI=[1.3–2.4], *P* = 4.6 × 10^− 4^), but was not significant for women with both prior and child/teen depression onset. Adverse mood effect was significantly associated with PGS for MD: full sample: RR = 1.18,CI=[1.1–1.3], *P* = 3.6 × 10^− 5^); no prior or child/teen depression onset: RR = 1.27,CI=[1.1–1.4], *P* = 3.1 × 10^− 4^.

**Conclusions:**

Participants who experience an adverse mood effect with OCP use are likely to have higher genetic vulnerability for depression, and experience child/teen depression onset, as well as reproductive depressive episodes such as PPD.

**Supplementary Information:**

The online version contains supplementary material available at 10.1007/s00737-026-01708-z.

## Highlights

Women who report OCP adverse mood effect show higher genetic vulnerability to major depression than those reporting neutral mood effect.

Women with adverse mood effect are significantly more likely to experience depression onset during childhood or teenage years than those with neutral mood effect.

More than half of those experiencing adverse mood effect had experienced at least one episode of depression prior to commencing an OCP.

A hormone-sensitive subset of women with no depression prior to commencing an OCP subsequently experienced either negative or positive mood effect and were significantly more likely to suffer from peripartum depression.

Women with PMDD were more likely to experience negative effect than neutral mood effect if they had also experienced prior depression, but positive mood effect if they had no prior depression.

**Supplementary Information:**

The online version contains supplementary material available at 10.1007/s00737-026-01708-z.

## Introduction

Depressive episodes are particularly common in women at times of change in reproductive hormones, including onset of menstruation, the peripartum period, and menopause, suggesting that abrupt changes in reproductive hormone levels can trigger depression in vulnerable women, called “reproductive depression” (Studd et al. 2012), or “reproductive related depressive episodes” (Soares 2019). These hormonal changes are a putative explanation for gender disparity in lifetime prevalence of depression after puberty (Abate 2013; Hodes et al. 2019, Trzaskowski et al. 2019), when it becomes approximately 50% more common amongst females than males (World Health Organisation 2023).

Reproductive related depressive episodes are thought to be due to hormone fluctuation (Mehta et al. 2019), irrespective of individual differences in absolute levels. The introduction of exogenous hormones through the use of oral contraceptives can establish stable hormone levels, which may improve symptoms of premenstrual dysphoric disorder (PMDD) (Nyberg 2013; de Wit et al. 2021), but may also promote adverse mood changes in vulnerable women.

A review of studies of psychiatric effects of the oral contraceptive pill (OCP) within the general population (Robakis et al. 2019) found inconsistent evidence, possibly due to confounding factors such as the omission of women who discontinued use due to adverse OCP mood effect (adverse mood effect), leading to healthy user bias (Johansson et al. 2023), or to the common practice of grouping women using different formulations of the OCP in a single cohort (Schaffir et al. 2016). Inconsistent findings may also reflect the complexity of depression, with many genetic variants and environmental influences contributing to risk. Other reviews (Fruzzetti et al. 2020, Sundström-Poromaa 2021; Mu et al. 2022, Noachtar et al. 2023) have reported risk of adverse mood effect for any formulation of the OCP, leading to the suggestion that a more effective approach might be to identify the subset of women at higher risk of developing mood disorders through OCP use (Fruzzetti et al. 2020). A relatively early study (Oinonen et al. 2002) reported increased likelihood of adverse mood effect in women with a prior history of depression, including a history of premenstrual and pregnancy-related mood symptoms. More recently, a large Danish study of women who had been diagnosed with depression before their first pregnancy (Larsen et al. 2023) reported that women who experienced their first episode of depression within 6 months of first use of an OCP (pill associated depression) were more likely to experience peripartum depression (PPD) than those with depression not associated with using an OCP. These results suggest the existence of a hormone sensitive subgroup of women who are more likely to experience depression due to exposure to an OCP, a conclusion supported by recent reviews of negative effect of OCP use (Sylvén et al. 2013; Fruzzetti et al. 2020).

If a hormone sensitive group does exist, it may in part be attributable to unique genetic influences relating to hormone sensitivity. It is therefore of interest to investigate how polygenic risk for depression differs between women experiencing adverse mood after taking the OCP and women who do not.

Our study was designed to answer the following questions:

1. Amongst women with lifetime depression, are those who report adverse mood effect through using an OCP more likely to experience reproductive related depressive episodes, such as PPD, PMDD, and perimenopausal depression, than those who do not report adverse mood effect?
2. Are there differences in vulnerability to depression, including genetic vulnerability, in those who report adverse mood effect compared to those who do not report adverse mood effect?

## Method

### Participants

The Australian Genetics of Depression Study, a case-cohort study of individuals who experience lifetime depression, recruited a total of 20,689 participants (75% female) primarily through a media campaign (86%). Full details of the recruitment strategy have been described in detail elsewhere (Byrne et al. 2020). The study sought participation from anyone who had been diagnosed with depression in their lifetime. Participants completed an online questionnaire (baseline), and those who consented to provide a DNA sample were mailed a saliva kit. In 2020, all participants who consented to be recontacted were invited to participate in a follow-up study. Consenting participants completed an online follow-up questionnaire that included questions about their use of OCPs. Participants who self-reported that they had ever taken the contraceptive pill were then asked ‘How does/did oral contraception affect your mood?’ (*no effect*, *improves it*, *makes it worse*). No information was gathered with respect to the particular formulation of OCP used, so it was not possible to test for an association between specific formulations of OCP and adverse mood effect. Participants in this study are females included in the 2018 data freeze (baseline), who reported OCP use, and its effect, in the follow-up questionnaire (*n* = 3,547).

### Depression cases

The baseline questionnaire included a compulsory module that assessed self-reported psychiatric history and clinical depression using the Composite Interview Diagnostic Interview Short Form (CIDI-SF) (Kessler et al. 1998). In addition, participants were asked whether they had ever been diagnosed, by a health professional, with a number of mental disorders, including depression, PPD, and PMDD. Female depression cases were women who self-reported a previous diagnosis of depression, or fulfilled DSM-IV (American Psychiatric Association 1994) criteria for MD. Parous women self-reporting symptoms of depression “around childbirth” were invited to complete the online self-report version of the Lifetime Edinburgh Postnatal Depression Scale (EPDS) (Cox et al. 1987; Meltzer-Brody et al. 2013). PPD cases (*n* = 1,318) had a score ≥ 13 on the Lifetime EPDS (Meltzer-Brody et al. 2013), or a previous diagnosis of PPD by a health professional, or reported a period of depression of at least 2 weeks that occurred in the peripartum period (during pregnancy or within six months of giving birth). PMDD cases (*n* = 122) self reported a previous diagnosis of PMDD. Perimenopausal cases (*n* = 69) were post-menopausal women who reported their age of depression onset to be within five years prior to their reported age of menopause.

### Association of OCP mood effect with patterns of OCP use

Age of first OCP use and number of years of use (independent variables) were tested for association with OCP mood effect (dependent variable with three categories: adverse mood effect; neutral OCP mood effect (neutral mood effect), positive OCP mood effect (positive mood effect)). The multinomial regression analysis used neutral mood effect as reference, and included age (as reported in the baseline questionnaire) as a covariate in all models.

### Association of OCP mood effect with peripartum depression, PMDD, perimenopausal depression, and depression onset

Multinomial regression tested the association of PPD, PMDD, and perimenopausal depression, as well as depression onset before starting an OCP (prior depression; *n* = 1,484), and depression onset before the age of twenty (childhood/teen depression onset; *n* = 2,041) (independent variables) with mood effect (dependent variable). For PPD, the number of births (exposures to risk of PPD) was included as a covariate. Since child/teen depression onset may be associated with increased likelihood of later episodes (Shorey et al. 2022), including an episode of PPD, it is a potentially confounding effect in any association of OCP adverse mood effect with reproductive depressive episodes. To test for independence, two sensitivity analyses were performed: the association of both PPD and PMDD with OCP mood effect was estimated in women with and without child/teen depression onset prior to commencement of an OCP, again adjusting for age (and, for PPD, number of births).

To run the multinomial regression analysis, the ‘multinom’ function in the R package ‘nnet’ (Venables et al. 2002) was used. The exponential of the effect size estimated by this function produces a relative risk ratio rather than an odds ratio. All regression estimates of effect were exponentiated to produce a relative risk ratio of either adverse or positive mood effect compared to neutral OCP mood effect, with a 95% confidence interval.

### Polygenic scoring

The influence of adverse life events on phenotypic indicators of depression vulnerability, such as child/teen depression onset, may complicate an association of phenotypic indicators with OCP adverse mood effect. Genetic vulnerability to depression, unaffected by such events, was measured using polygenic scores (PGS). SBayesRC (Zheng et al. 2022) was applied to a recent MD GWAS with Australian samples removed (Howard et al. 2019) to generate SNP weights, which were used with Plink 2.0 to generate an MD PGS for each participant with available genetic information (*n* = 3,421). SBayesRC is a Bayesian method that reweights the SNP effects reported in the GWAS summary statistics, using the assumption that these SNP effects are drawn from a mixture of distributions dependent on effect sizes and biological functions of different genomic regions with membership probabilities directly estimated. The set of individual MD PGS were standardized using the mean and standard deviation of the neutral mood effect subgroup. Multinomial regression was then used to regress OCP mood effect on MD PGS, including both age and age of depression onset as covariates, to test whether women reporting adverse or positive mood effect vary in genetic vulnerability to depression compared to women who reported no OCP mood effect. Finally, a sensitivity analysis tested the association of OCP mood effect with MD PGS for women who had not experienced prior or child/teen depression onset, also adjusting for age and age of depression onset.

### Correction for multiple testing

**Fig. 1:**
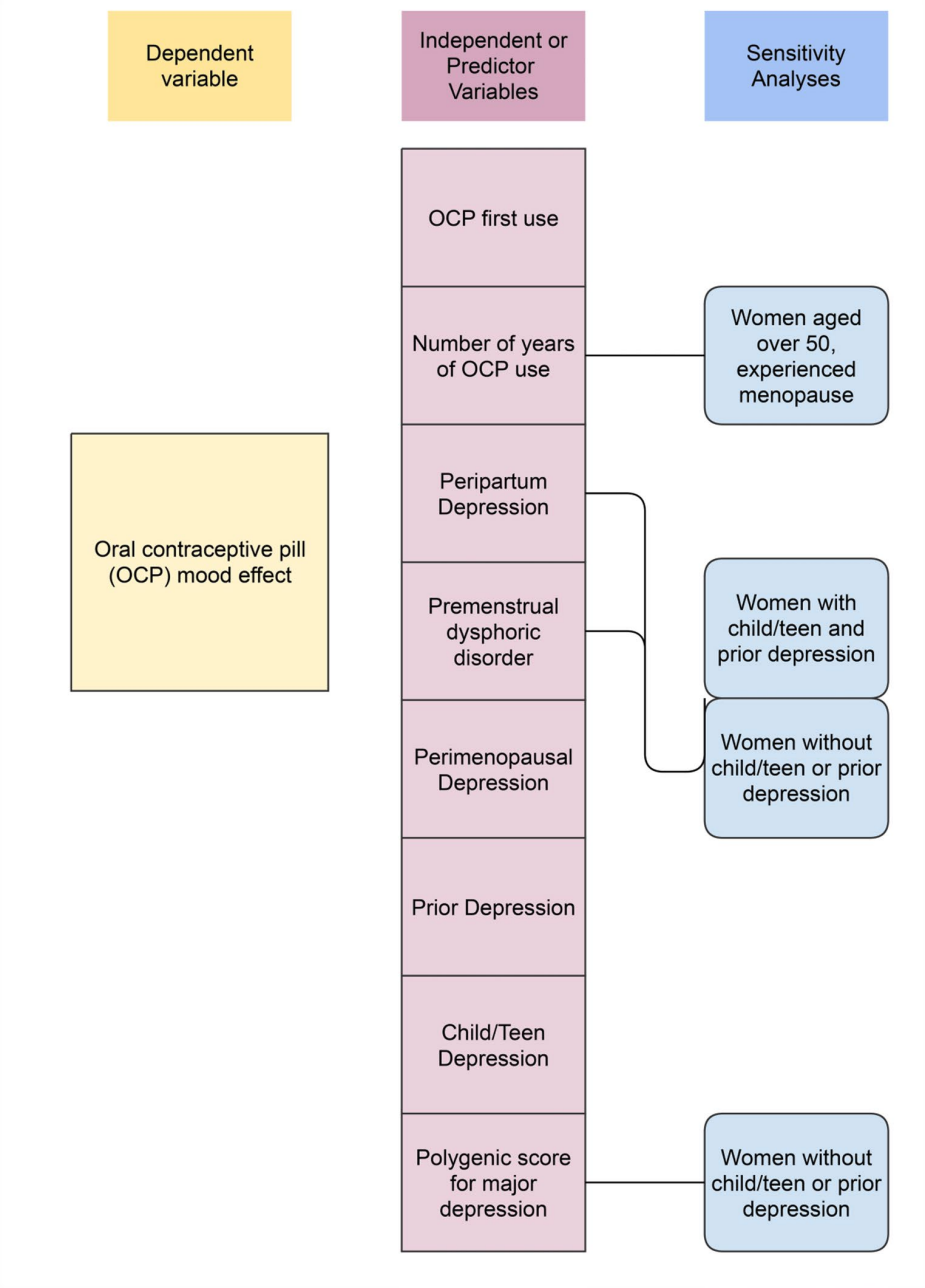
Association analyses: The dependent variable (OCP mood effect) was regressed on eight independent variables. Sensitivity analyses tested association of variables within the group of women with or without child/teen depression onset and depression prior to OCP commencement

A total of eight independent multinomial regression models and four correlated sensitivity models were constructed, described above, and summarised in Fig. 1. Each model comprised two comparisons: negative and positive mood effect compared to no effect. All p-values were adjusted for multiple testing, using the conservative Bonferroni method (*n* = 16; alpha 0.05/16 = 0.003).

## Results

### Sample demographics and patterns of OCP use

To test whether follow-up participants were representative of baseline participants, the distributions of age, ancestry, and educational attainment, as well as severity of depression (measured using number of episodes, number of symptoms, and length of “worst” depression episode) were compared for all female participants across baseline (*n* = 15,516) and follow-up (*n* = 6,572) data. Results, provided in Supplementary Information, with details of ancestry provided in Table S1, indicated that participants who completed the follow-up questionnaire did not differ significantly from those who completed the original AGDS questionnaire. To summarize, the Australian Genetics of Depression Study, both at baseline and follow-up, comprises predominantly European and highly educated participants who have experienced multiple episodes of depression in their lifetime.

Of 5,631 women who answered the question about OCP use, 5,168 (91.8%) self-reported using an OCP, although only 3,547 answered the question about the effect of OCP use on their mood. A comparison of sociodemographic and clinical variables across the two groups indicated that respondents were significantly younger and more highly educated than non-respondents, but there was no significant difference in reported experience of depression (Table 1).

**Table 1:** A comparison of respondents (provided information about OCP mood effect) and non-respondents

Variable	Statistic	Respondents	Non-respondents	Signficance
Age	Mean (SD)	43.3 (14.8)	45.2 (14.4)	t-test=-4.47; *P* < 0.001
Education	% degree or postgraduate studies	66.8	61.8	$\:\chi\:$=12.08; *P* < 0.001
Number depression symptoms	% 8 + symptoms	80	79.5	$\:\chi\:$ =0.12; *P* = 0.73
Number depression episodes	% 7 + episodes	99.9	99.9	$\:\chi\:$=0.67; *P* = 0.41
Length worst depression episode	% > 3 months	43.7	43.8	$\:\chi\:$=0.0; *P* = 0.99
Depression onset	% child or teen onset	61.7	59.8	$\:\chi\:$=1.42; *P* = 0.23
Peripartum depression (PPD)	% experienced PPD	37.2	39.2	$\:\chi\:$ =1.84; *P* = 0.18

Of women who provided information about the effect of OCP use on their mood, approximately half responded that OCPs had no effect (*n* = 1,826, 51%), 379 (11%) responded that OCPs improves their mood and 1,342 (38%) reported worsened mood. To test for possible difference in mood effect for different generations of OCP, women were grouped into cohorts, each spanning one decade, according to the year in which they first used an OCP (Table 2). As illustrated in Table 2, all generations of OCP in use across the decades carry a risk of adverse mood effect.

**Table 2:** Effect of OCP on mood according to decade of initial OCP use

Decade	Effect of OCP on mood			Total number
	No effect	Improves it	Makes it worse	
< 1961	1 (50%)		1 (50%)	2
1961–1970	106 (65%)	16 (10%)	42 (26%)	164
1971–1980	436 (65%)	68 (10%)	171 (25%)	675
1981–1990	412 (60%)	77 (11%)	203 (29%)	692
1991–2000	330 (46%)	81 (11%)	303 (42%)	714
2001–2010	345 (40%)	94 (11%)	414 (49%)	853
2011–2018	193 (44%)	41 (9%)	206 (47%)	440

### Patterns of OCP usage

For all analyses reported in this, and following, sections, details of results are provided in Table S2.

**Fig. 2:**
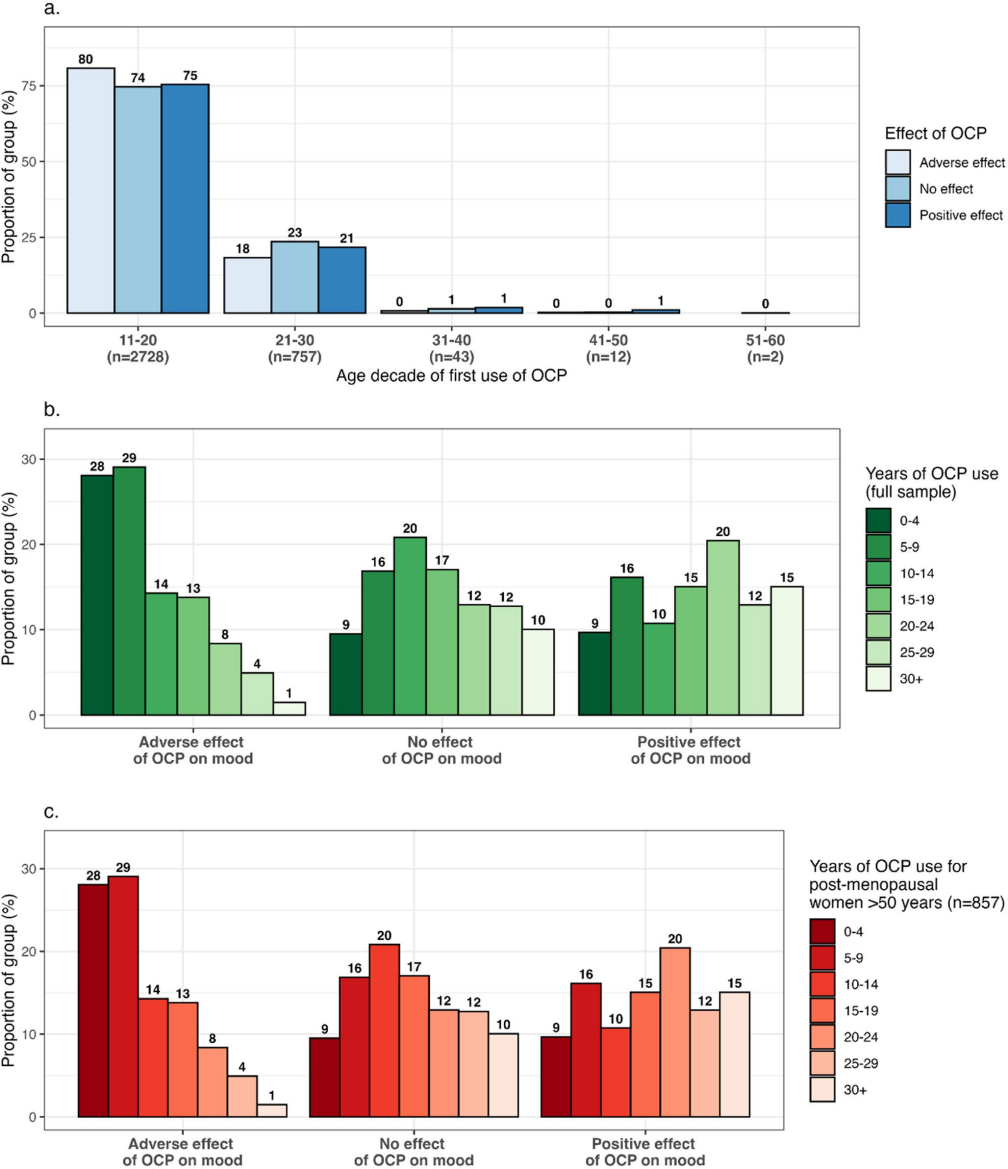
Patterns of oral contraceptive pill (OCP) use according to its effect on mood: **a** Age of first use of an OCP (**b**) Years spanning OCP use (full sample) (**c**) Years spanning OCP use for the subset of women who had experienced menopause and who reported an age > 50 years in the baseline questionnaire

Patterns of OCP usage, illustrated in Fig. 2, demonstrates the relationship of OCP effect on mood with both the age of first use of an OCP and the years spanning OCP usage (calculated by subtracting the age of first use from the age of last use). Overall, 2,728 women (77.0%) reported first OCP use before age 20 (80.7% of the adverse mood effect group, 74.6% of the neutral mood effect group and 75.4% of the positive mood effect group). Only 57 women (1.6%) reported first use of an OCP after age 30. There was no significant difference in the age of first use of an OCP across its effect on mood after correcting for age at completion of questionnaire (Fig. 2A, Table S2). However, as might be expected, the effect of the OCP on mood was significantly negatively correlated with the number of years of OCP usage, with 28.4% of those who experienced adverse mood effect discontinuing use within five years and 56.3% within ten years, compared to 13.2% and 32.5% respectively of those who experienced neutral mood effect, and 10.8% and 28.7% of those who experienced positive mood effect (Fig. 2B). The regression of OCP mood effect on years of OCP use as a continuous measure reported that the relative risk of an adverse, compared to a neutral, effect was 0.94 per additional year of OCP use (CI=[0.93–0.95], *P* = 7.0 × 10^− 28^), whilst the relative risk of a positive, compared to a neutral mood effect was 1.03 per additional year of OCP use (CI=[1.01–1.04], *P* = 1.1 × 10^− 03^) (Table S2) after correcting for age, which was also significantly associated with years of OCP use (*P* = 1.3 × 10^− 27^). Since factors such as the age of participant, or temporary OCP interruption in order to have children (coinciding with questionnaire completion), might confound this result, a sensitivity analysis was conducted of women who had experienced menopause and were more than 50 years of age on completion of the baseline questionnaire (*n* = 857). For this subset, 28.1% of those who experienced adverse mood effect reported that they had stopped using an OCP within five years, and 57.2% within ten years, compared to 9.5% and 16.9% respectively of those who experienced neutral mood effect (Fig. 2C) (RR[adverse mood] = 0.93 per additional year of OCP use, CI=[0.91–0.94], *P* = 1.1 × 10^− 13^). Positive mood effect was no longer significantly associated with years spanning OCP use for this subset (*P* = 8.4 × 10^− 2^).

### Association of adverse mood effect with depression

Among those reporting adverse mood effect from the OCP, 40.4% met criteria for PPD, compared to 37.7% of the positive mood effect group, and 34.7% of the neutral mood effect group. The relative risk of adverse mood effect for a woman who had experienced an episode of PPD, compared to the relative risk of neutral mood effect, was 1.66 (CI=[1.35–2.04], *P* = 2.0 × 10^− 6^, significant after Bonferroni correction (Table 3). This means that women who have experienced PPD are 66% more likely to report adverse mood effect from the pill rather than neutral mood effect, relative to women without a PPD history. Unexpectedly, the relative risk of positive mood effect for PPD cases compared to the relative risk of neutral mood effect was also significant, and carried a higher risk ratio (RR = 1.83, CI=[1.32–2.56], *P* = 3.5 × 10^− 4^). These results suggest that women with a history of PPD are more likely to have non-neutral mood responses (both negative and positive) to OCPs than women without PPD.

A total of 122 participants reported being diagnosed with PMDD. Of these, 67 reported adverse mood effect (55%), 29 (24%) reported positive mood effect, and 26 (21%) PMDD cases experienced neutral mood effect. These differences were statistically significant. For PMDD cases, the relative risk of an adverse mood effect, compared to a neutral mood effect, was 3.78 (CI=[2.37–6.03], *P* = 2.2 × 10^− 8^) and 5.82 (CI=[3.38-10.00], *P* = 2.1 × 10^− 10^) for a positive mood effect (Table 3).

**Table 3:** Association of OCP mood effect with PPD and PMDD

Association with Depression	Group in focus	Number of cases (%) in focus group	Number of cases (%) in neutral mood effect group	Relative Risk Ratio	Confidence interval (95%)	*P*
PPD	Adverse mood effect	542 (40.4)	633 (34.7)	1.66	1.35–2.04	2.0 × 10^− 6^
PPD	Positive mood effect	143 (37.7)	633 (34.7)	1.83	1.32–2.56	3.5 × 10^− 4^
PMDD	Adverse mood effect	67 (5)	26 (1.4)	3.78	2.37–6.03	2.2 × 10^− 8^
PMDD	Positive mood effect	29 (7.7)	26 (1.4)	5.82	3.38-10.00	2.1 × 10^− 10^

Regression analysis indicated that women who experienced their first episode of depression in the perimenopausal period were less likely to have experienced adverse mood effect or positive mood effect than neutral mood effect (with a relative risk ratio of 0.66 and 0.72 respectively), but neither of these results were significant (Table S2).

**Fig. 3:**
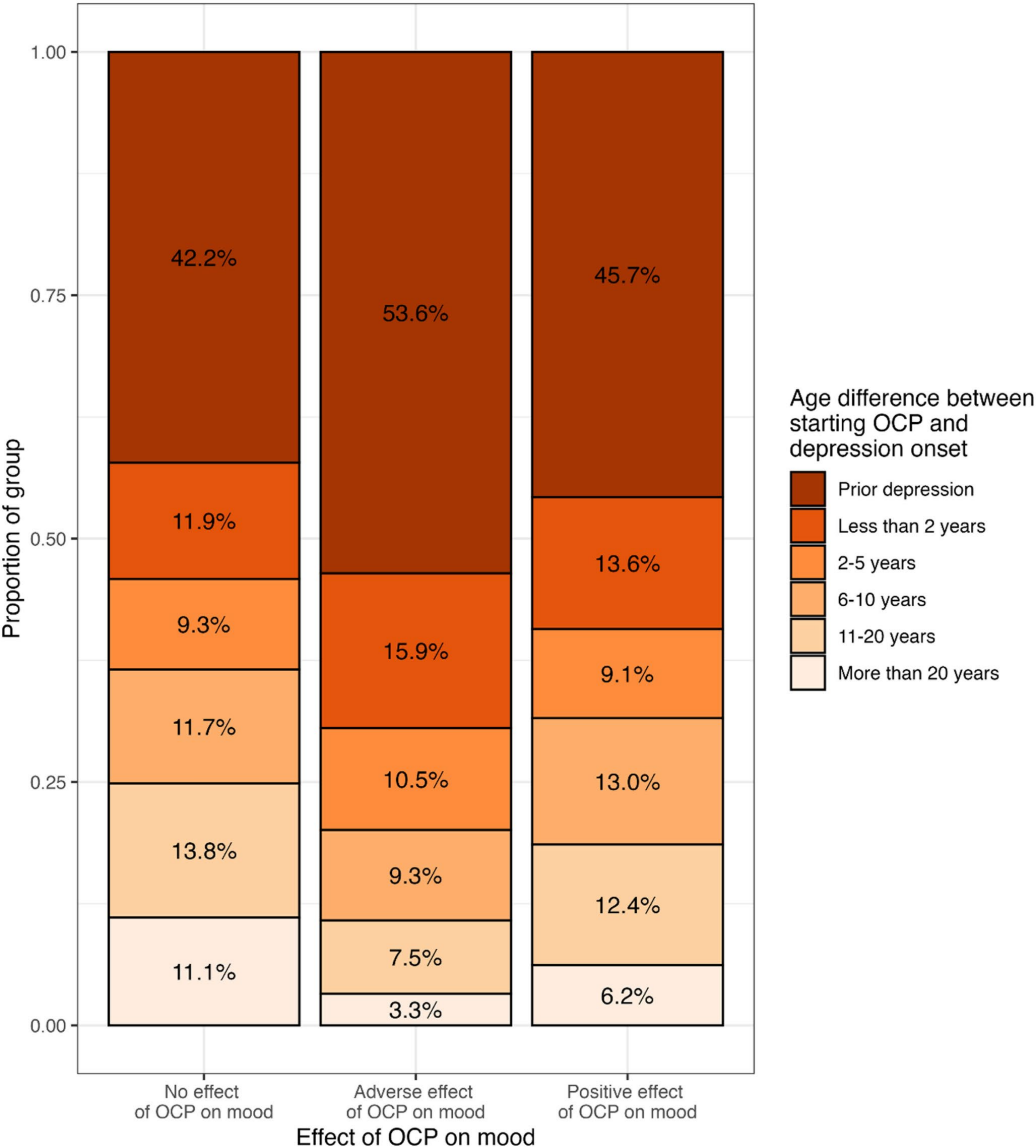
Relationship between oral contraceptive pill (OCP) mood effect and depression onset, according to the age difference between starting an OCP and depression onset

Figure 3 illustrates the relationship between OCP mood effect and the age difference between starting an OCP and depression onset. An episode of depression prior to commencing an OCP was reported by 53.6%, 45.7% and 42.2% of those reporting adverse, positive and neutral mood effect respectively. Compared to the likelihood of neutral mood effect, onset of depression prior to starting an OCP was significantly associated with increased likelihood of reporting adverse mood effect (RR = 1.32, CI=[1.13–1.54], *P* = 5.9 × 10^− 4^) after correction for age. The association between positive mood effect and prior depression was not significant (*P* = 5.3 × 10^− 1^).

Given that those with adverse mood effect are likely to have early depression onset, we sought to investigate whether age of depression onset was driving the significant associations between adverse mood effect and reproductive depressive episodes. We conducted a sensitivity analysis where the association between PPD, PMDD and OCP mood effect was evaluated only in women who reported not having a depressive episode prior to OCP commencement and whose first depressive episode occurred after the age of nineteen (*n* = 1,184). Within this group, PPD cases (*n* = 527) comprised 48%, 47% and 38% percent of those who had experienced adverse, positive or neutral mood effect respectively (RR[adverse mood effect] = 1.77, CI=[1.29–2.43], *P* = 4.6 × 10^− 4^; RR[positive mood effect] = 1.99, CI=[1.23–3.21], *P* = 4.9 × 10^− 3^ after correcting for age and number of births). The sample of women who met criteria for PMDD and who had onset of depression after taking OCP, numbering 11, 12, and 8 for adverse, positive, and neutral mood effect respectively, was too small to reach significance.

We next tested the association of PPD and PMDD with OCP mood effect in the subset of women who reported at least one episode of depression prior to OCP commencement as well as child/teen depression onset (*n* = 1,394). Amongst this subset, the association between PPD and OCP mood effect was not significant, with p-values of 2.18 × 10^− 1^and 2.3 × 10^− 1^ for adverse and positive mood effect respectively. The association between PMDD and adverse mood effect remained significant for women with prior and child/teen depression (RR = 4.27, CI=[1.68–10.8], *P* = 2.2 × 10^− 3^), although small sample sizes incurred large confidence intervals. The nominally significant association between PMDD and positive mood effect for this sample did not survive Bonferroni correction (Table S2).

### Association of OCP mood effect with PGS for MD

**Fig. 4:**
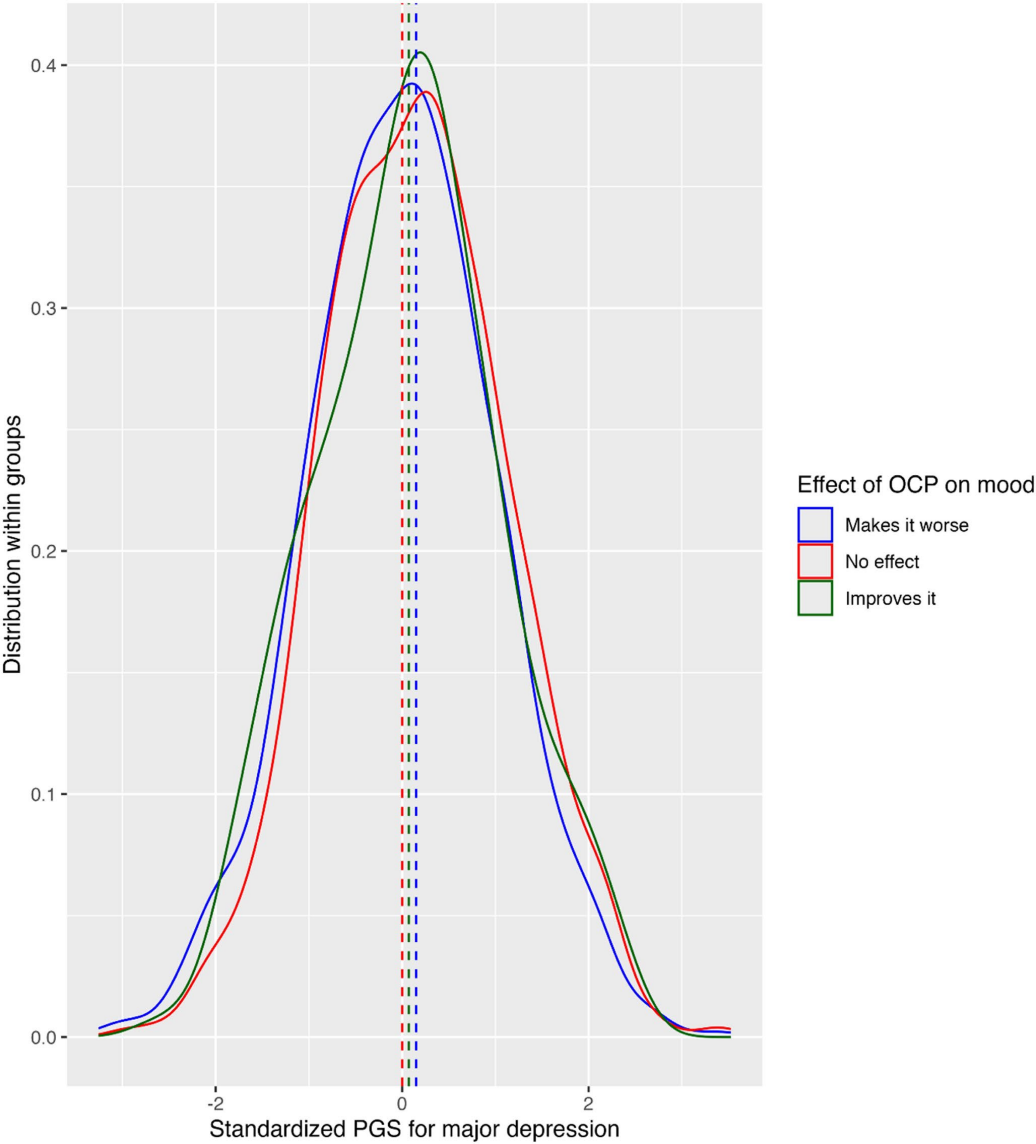
Density plot of polygenic scores (PGS) for major depression, grouped according to reported effect on mood of oral contraceptive pill (OCP)

Difference in standardized MD PGS for women who used an OCP, according to OCP mood effect, is illustrated in Fig. 4. The standardized means for the adverse mood effect group (0.15) and the positive mood effect group (0.07) were both higher than that of the neutral mood effect group. The difference between the adverse and neutral mood effect groups was significant: the linear regression model indicated that the relative risk of adverse mood effect compared to the relative risk of neutral mood effect was 1.18 per standard deviation increase in MD PGS (CI=[ 1.09–1.27], *P* = 3.6 × 10^− 5^) after correcting for both age and age of depression onset; Cohen’s d = 0.15 (CI=[0.08–0.22]). MD PGS was not significantly associated with positive mood effect. MD PGS also predicted adverse mood effect for participants who had not experienced depression before OCP use or before the age of twenty, with a relative risk ratio of 1.26 per standard deviation increase in standardized scores (CI=[1.11–1.43], *P* = 4.2 × 10^− 4^) after correcting for both age and age of depression onset; Cohen’s d = 0.23 (CI=[0.10–0.36]).

## Discussion

In this study of women with depression, self-reported adverse mood effect as a result of using an OCP was experienced by almost 40% of the sample and positive mood effect was experienced by just 11%, but both these effects were significantly associated with experience of reproductive depression in the form of PPD and PMDD. Since reproductive depression is thought to be stimulated by sensitivity to fluctuations in hormone levels, the stabilizing influence of an OCP may promote positive mood change. PMDD cases in this study may have chosen to use an OCP for this purpose, although a recent review of treatments for PMDD (Nappi et al. 2022) noted that the effect of an OCP on mood is “highly variable”, with a generally poor response for women with a history of mood affective disorders. This finding is supported by the present study, where a significant association of positive mood effect with PMDD only exists amongst women with no child/teen onset or depression onset before commencing an OCP. For women who had experienced at least one depression episode before the age of twenty and before commencing an OCP, PMDD was significantly associated with adverse mood effect.

The association between using an OCP and depression has been the focus of much research e.g. (Rohleder et al. 2003; Skovlund et al. 2016; Bitzer 2017; Robakis et al. 2017, 2019; Fruzzetti et al. 2020, Larsen et al. 2020; Gervasio et al. 2022; Johansson et al. 2023; Larsen et al. 2023). Whether a causal influence exists remains contentious, although associations of oral contraception with changes in serotonin signalling (Larsen et al. 2020), in cortisol levels (Rohleder et al. 2003; Gervasio et al. 2022), and with de novo methylation events (Moradi Sarabi et al. 2017) have been cited as possible mechanistic pathways leading to depression onset. However, the present study found that, compared to those experiencing neutral adverse mood effect, a significantly higher proportion of those who experienced adverse mood effect had onset of depression before the age of twenty. Our study indicates that participants who experience adverse mood effect from OCPs have greater genetic vulnerability for depression, and more than half of them have already experienced at least one severe depressive episode prior to OCP use. The results from the PGS analysis further supported the hypothesis that adverse mood effect may imply a risk of more severe depression, as those reporting adverse mood effect had a significantly higher polygenic risk for MD. In addition, amongst women with depression onset before the age of twenty, or prior to OCP commencement, OCP mood effect was no longer significantly associated with PPD, indicating that depression vulnerability confounds the association of PPD with both adverse and positive mood effect.

The association between PPD and adverse mood effect remained significant for women who did not suffer child/teen depression onset, or any depression episode before commencing an OCP. This group may form a hormone-sensitive subset of women, who have not experienced depression before OCP use, but are subsequently likely to suffer both OCP adverse mood effect and PPD. In addition, the significant association of PMDD with adverse mood effect for the child/teen and prior depression group suggests a complexity in ‘hormone sensitivity’ that spans both early and late depression onset. Genetic multivariate analysis conducted in an early twin study (Kendler et al. 1988) suggested that the genes underlying OCP induced depression may be distinct from MD occurring without pharmacological agents (Kendler et al. 1988). Our study supports the notion of heterogeneity within MD, to which hormone sensitivity, with unique genetic influences, makes a significant and complex contribution. This greater variability or sensitivity may reflect underlying neurobiological differences in OCP hormone response among women who experience PPD, which manifest in either adverse or positive mood effect.

This study has some important limitations. Firstly, no information was available with respect to the type and concentration of OCP that was used by each participant. Given the several different types of OCP, containing different formulations of estrogen, including none at all (progestin only pill or mini pill), this lack of information was a serious limitation on the analysis of mood effect, and reflected the nature of the data, based on an online questionnaire, with no personalized interviews or clinical reports to provide supporting evidence for self-reported data. No information was obtained with respect to whether participants were using other medications or supplements that may affect use of an OCP, or their purpose in using an OCP, which may not always be prescribed for contraception. Indeed, PMDD cases within the present study who reported positive mood effect may have been using an OCP for mood stabilisation. Whilst the study design circumvented these limitations through a focus on the subset of women who are vulnerable to adverse mood effect of any OCP formulation, the effects of specific OCP formulations on mood could not be estimated, limiting the applicability of results. The increased prevalence of adverse mood effect for those whose initial OCP use occurred post 1990 may reflect the introduction of 3rd and 4th generation OCP’s during the 1990’s, although further analysis of the pre- and post1990 groups suggested that a more likely explanation is a healthy older participant bias (Supplementary Information). However, without details of the specific OCP used, explanations remain tentative.

Secondly, this was a retrospective study and so information on the timing of depression onset in relation to the taking of the OCP is dependent upon the accuracy of recall of the participants. There is a clear need for prospective studies that assess mental health symptoms from prior to initiating the OCP until many decades later to better evaluate the links between OCP use and reproductive timing of depressive episodes.

The sample also suffered from ascertainment bias, given the prevalence of a high level of education amongst participants, who were also predominantly of European ancestry, so that results may not reflect those of the wider population. Although the advantage of the online questionnaire format is the consequent large sample size, some associations were still limited by small sample sizes, particularly the association of adverse mood effect with PMDD, for which there were only 122 reported cases, resulting in large confidence intervals. Nevertheless, our data provide substantiation of the association between hormonal or reproductive related depressive episodes such as PPD and PMDD, and both adverse and positive mood effect from OCP use, including evidence of the confounding influence of a previous history of depression on these associations. In providing evidence that those experiencing adverse mood effect have a higher vulnerability for depression, including genetic vulnerability, this study highlights cases where OCP use may not be best practice. Since almost 30% of participants in this study who experienced adverse mood effect had stopped using an OCP within five years, compared to 13% of participants who experienced either a neutral or positive mood effect, our results also support the suggestion that contraceptive counselling and care should be tailored to the individual (Bitzer 2017).

## Electronic Supplementary Material

Below is the link to the electronic supplementary material.

Supplementary Material 1

## Data Availability

No datasets were generated during the current study.
