# Supplementary material for: Poor mood after oral contraceptive use is associated with increased vulnerability to peripartum depression, premenstrual dysphoric disorder, and higher genetic risk for depression
Source: Arch Womens Ment Health. 2026 May 6;29(3):71. doi: 10.1007/s00737-026-01708-z (PMC13149705; doi:10.1007/s00737-026-01708-z)
Supplement: Supplementary file 1 — Supplementary Material 1 [file 737_2026_1708_MOESM1_ESM.docx]

Poor mood after oral contraceptive use is associated with increased vulnerability to peripartum depression, premenstrual dysphoric disorder, and higher genetic risk for depression

Supplementary Information

Table of Contents

[Comparison of baseline and follow-up study participants 2](#_Toc216906888)

[Comparison of participants according to their initial use of an OCP 3](#_Toc216906889)

[Supplementary Figures 4](#_Toc216906890)

[Figure S1. Comparison of female baseline (n=15,516) and follow-up (n=7,133) data across age, ancestry and educational achievement 4](#_Toc216906891)

[Figure S2. Comparison of female baseline (n=15,516) and follow-up (n=7,133) data for severity of depression 5](#_Toc216906892)

[Figure S3. Forest plot: Risk ratio of peripartum depression (PPD) and premenstrual dysphoric disorder (PMDD), given OCP mood effect, according to first OCP use 6](#_Toc216906893)

[Supplementary Tables 7](#_Toc216906894)

[Table S1. Geographical region of ancestors 7](#_Toc216906895)

[Table S2. Logistic regression results 8](#_Toc216906896)

[Table S3. Logistic regression results for those whose first use of an OCP occurred prior to 1990 11](#_Toc216906897)

[Table S4. Logistic regression results for those whose first use of an OCP occurred post 1990 13](#_Toc216906898)

### Comparison of baseline and follow-up study participants

To test whether follow-up participants were representative of baseline participants, the distributions of age, ancestry, and educational attainment, as well as severity of depression (measured using number of episodes, number of symptoms, and length of “worst” depression episode) were compared for all female participants across baseline (n=15,516) and follow-up (n=6,572) data. Results, illustrated in Supplementary Figures 1 and 2, with details of ancestry provided in Table S1, indicate broad correspondence between baseline and follow-up sample groups. The mean age at baseline was 41.2 (range = 18-90), with a mode of 27, and the mean age at follow-up was 43.4 (range = 18-90), also with a mode of 27. Educational attainment was high for both baseline and follow-up groups, with completion or partial completion of a degree reported by the highest proportion of participants (baseline: 33%, follow-up: 35%), followed by post-graduate studies (baseline: 24%, follow-up: 29%). Both baseline and follow-up sample groups were predominantly European, with 80% of the baseline sample and 83% of the follow-up sample groups reporting United Kingdom ancestry (Table S1). For both baseline and follow-up sample groups, distribution of depression episodes peaked at 13 or more episodes (34% of participants for both groups), and the distribution of depression symptoms peaked at the maximum nine symptoms (baseline: 53%, follow-up: 51%). The length of depression also followed a similar distribution for both groups, with one to three months most commonly reported (baseline: 36%, follow-up: 37%). To summarize, the Australian Genetics of Depression Study, both at baseline and follow-up, comprise participants who are relatively young, predominantly European, and highly educated, and who have experienced many episodes of depression in their lifetime.

### Comparison of participants according to their initial use of an OCP

Initial OCP use in the decades post 1990 show increased prevalence of adverse OCP mood effect compared to previous decades (Table 2). To investigate whether this increase in prevalence reflects the introduction of 3^rd^ and 4^th^ generation OCPs in the 1990’s, a secondary analysis tested the association of PPD, PMDD, perimenopausal depression, prior depression and childhood/teen depression onset with mood effect for each of two groups: those whose initial OCP use occurred prior to 1990 (prior1990) and those whose initial OCP use occurred during or after 1990 (post1990). Key results are illustrated in Figure S3, with full details provided in Tables S3 and S4. As illustrated in Figure S3, results do not vary markedly across the groups, and an alternative explanation for the increased prevalence is that of healthy older participant bias. The post1990 group has a mean age of 32.5 and mean age of depression onset of 17.8 compared to 57.5 and 25.8 respectively for the prior1990 group, with implications for associated OCP mood effects.

### Supplementary Figures


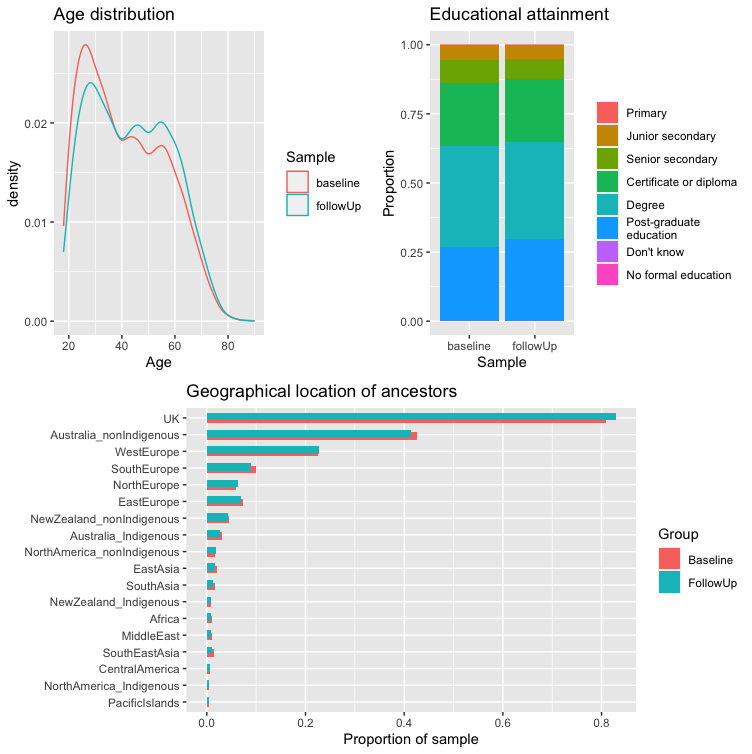


Figure S1. Comparison of female baseline (n=15,516) and follow-up (n=7,133) data across age, ancestry and educational achievement. The data represents a relatively young, overwhelmingly European, and highly educated sample population. According to these demographics, the participants who volunteered for the follow-up survey are representative of all participants in the baseline survey of the Australian Genetics of Depression Study.


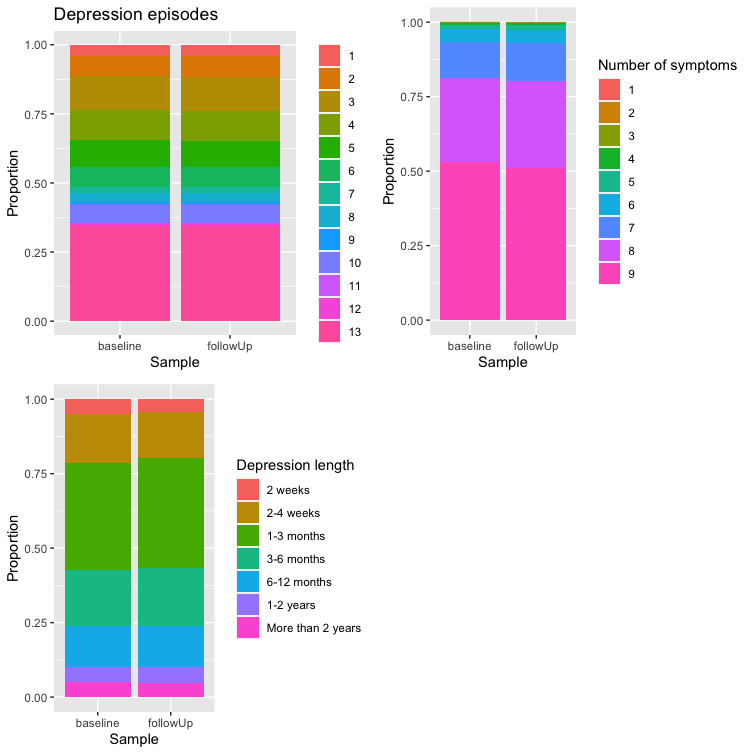


Figure S2. Comparison of female baseline (n=15,516) and follow-up (n=7,133) data for severity of depression, measured using number of episodes, number of symptoms and depression length. The data represents a severely depressed sample population. In terms of depression severity, the participants who volunteered for the follow-up survey are representative of all participants in the baseline survey of the Australian Genetics of Depression Study.


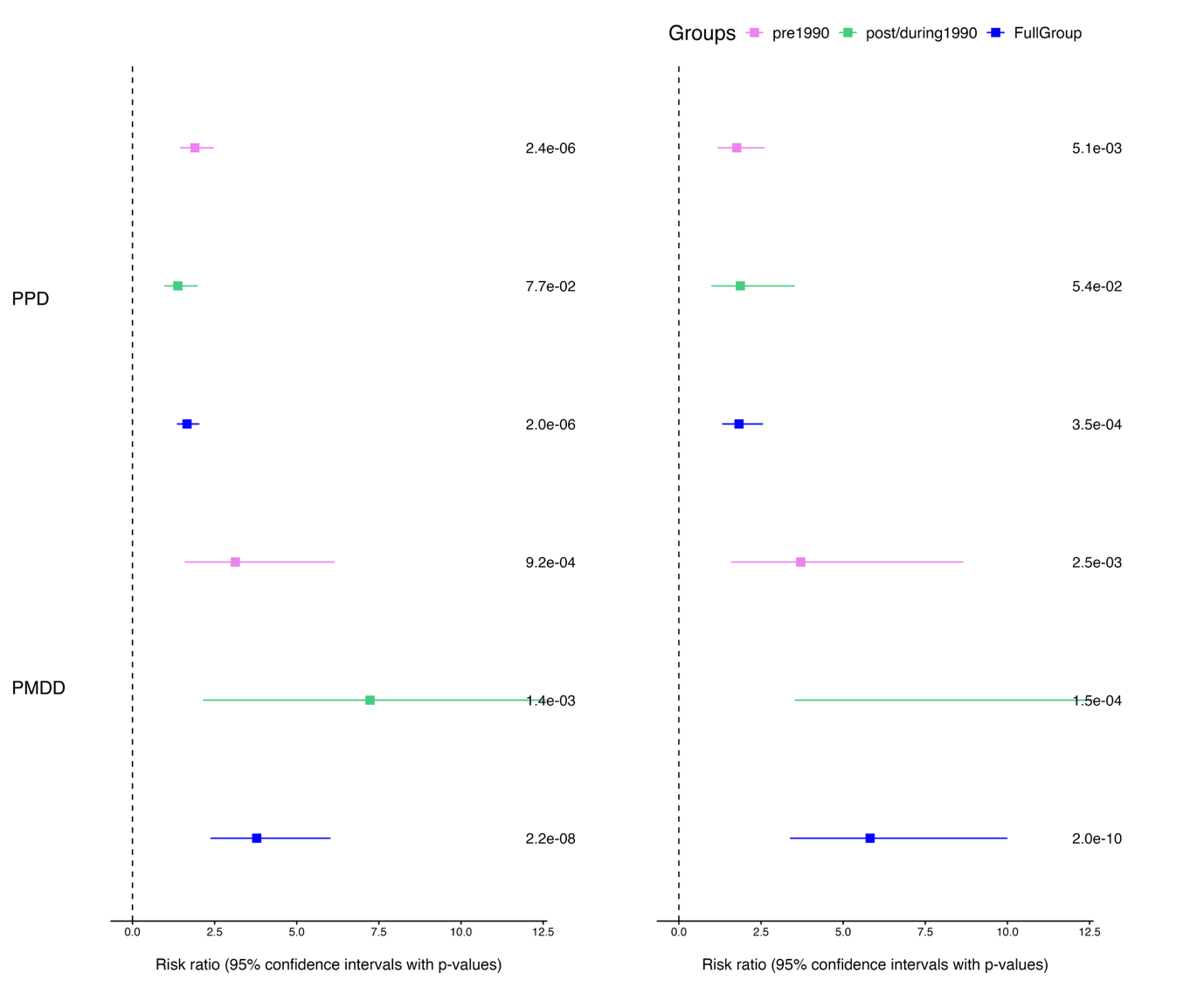


Figure S3. Forest plot: Risk ratio of peripartum depression (PPD) and premenstrual dysphoric disorder (PMDD), given OCP mood effect, according to first OCP use: those whose first use of an OCP was in or before 1990; those whose first use was post 1990, and both groups (full group). Left: adverse mood effect; right: positive mood effect.

### Supplementary Tables

Table S1. Geographical region of ancestors (e.g. great great grandparents) for 1. baseline - all female participants; 2. follow-up - all female participants; 3. follow-up - sample of oral contraceptive users. Participants may have nominated more than one region.

| Geographical region | Proportion (Baseline: all females, n=13,596) | Proportion (Follow up: all females, n=6,368) | Proportion (Sample: n=3,434) |
| --- | --- | --- | --- |
| England, Ireland, Scotland or Wales | 0.81 | 0.83 | 0.84 |
| Australia - not of Aboriginal or Torres Strait Islander descent | 0.43 | 0.41 | 0.41 |
| Western Europe including France, Germany, the Netherlands and surrounding countries | 0.22 | 0.23 | 0.23 |
| Southern Europe including Italy, Greece, Spain, Portugal and surrounding countries | 0.10 | 0.09 | 0.09 |
| Northern Europe including Sweden, Norway, Finland and surrounding countries | 0.06 | 0.06 | 0.07 |
| Eastern Europe including Russia, Poland, Hungary and surrounding countries | 0.07 | 0.07 | 0.06 |
| NewZealand - not of Maori descent | 0.04 | 0.04 | 0.04 |
| Australia - of Aboriginal or Torres Strait Islander descent | 0.03 | 0.03 | 0.03 |
| North America - not of First Nations, Native American, inuit or Métis descent | 0.02 | 0.02 | 0.02 |
| Eastern Asia including China, Japan, South Korea, North Korea, Taiwan and Hong Kong | 0.02 | 0.02 | 0.02 |
| South Asia including India, Pakistan, Sri Lanka and surrounding countries | 0.02 | 0.01 | 0.01 |
| South-East Asia including Thailand, Malaysia, Indonesia, Singapore and surrounding countries | 0.01 | 0.01 | 0.01 |
| Africa | 0.01 | 0.01 | 0.01 |
| Middle East including Lebanon, Turkey and surrounding countries | 0.01 | 0.01 | 0.01 |
| New Zealand Indigenous | 0.01 | 0.01 | 0.01 |
| Caribbean, Central or South America | 0.01 | 0.01 | 0.00 |
| North America - of First Nations, Native American, inuit or Métis descent | 0.00 | 0.00 | 0.00 |
| Polynesia, Micronesia or Melanesia including Tonga, Fiji, Papua New Guinea and surrounding countries | 0.01 | 0.00 | 0.00 |
| Only European ancestry | 0.49 | 0.52 | 0.52 |
| No reported European ancestry | 0.03 | 0.02 | 0.02 |

Table S2. Logistic regression results: Patterns of oral contraceptive pill (OCP) use; association of OCP adverse or positive mood effect with depression and polygenic risk scores for major depression (MD).

| **Patterns of OCP use** | Group compared with 'No effect' | Relative risk ratio | | CI | | P | | adjusted P (Bonferroni) | |  |  |  |  |
| --- | --- | --- | --- | --- | --- | --- | --- | --- | --- | --- | --- | --- | --- |
| Age of first use of OCP | Adverse effect | 0.99 per additional year of age | | 0.97-1.00 | | 1.19E-01 | | 1.00 | |  |  |  |  |
|  | Positive effect | 1.01 per additional year of age | | 0.98-1.03 | | 5.51E-01 | | 1.00 | |  |  |  |  |
| Years spanning usage (full group) | Adverse effect | 0.94 per additional year of use | | 0.93-0.95 | | 7.02E-28 | | 0.00 | |  |  |  |  |
|  | Positive effect | 1.03 per additional year of use | | 1.01-1.04 | | 1.10E-03 | | 0.02 | |  |  |  |  |
| Years spanning usage (women >50 who had experienced menopause) | Adverse effect | 0.93 per additional year of use | | 0.91-0.94 | | 1.07E-13 | | 0.00 | |  |  |  |  |
|  | Positive effect | 1.02 per additional year of use | | 1.00-1.04 | | 8.35E-02 | | 1.00 | |  |  |  |  |
| **Association with depression** |  | |  | |  | |  | |  | |  |  |  |
|  | Num (%) of OCP adverse effect group (n=1,342) | | Num (%) of OCP positive effect group (n=379) | | Num (%) of OCP no effect group (n=1826) | | Group compared with 'No effect' | | Relative risk ratio | | CI | P | adjusted P (Bonferroni) |
| Peripartum depression (PPD) | 542 (40.4) | | 143 (37.7) | | 633 (34.7) | | Adverse effect | | 1.66 | | 1.35-2.04 | 1.97E-06 | 0.00 |
|  |  | |  | |  | | Positive effect | | 1.83 | | 1.32-2.56 | 3.50E-04 | 0.01 |
| Premenstrual dysphoric disorder (PMDD) | 67 (5) | | 29 (7.7) | | 26 (1.4) | | Adverse effect | | 3.78 | | 2.37-6.03 | 2.22E-08 | 0.00 |
|  |  | |  | |  | | Positive effect | | 5.82 | | 3.38-10.00 | 2.05E-10 | 0.00 |
| Perimenopausal depression | 13 (5.9) | | 6 (6.3) | | 50 (9) | | Adverse effect | | 0.66 | | 0.35-1.23 | 1.90E-01 | 1.00 |
|  |  | |  | |  | | Positive effect | | 0.72 | | 0.30-1.73 | 4.57E-01 | 1.00 |
| Depression before starting OCP (prior depression) | 640 (53.6) | | 155 (45.7) | | 689 (42.4) | | Adverse effect | | 1.32 | | 1.13-1.54 | 5.90E-04 | 0.01 |
|  |  | |  | |  | | Positive effect | | 1.08 | | 0.85-1.38 | 5.26E-01 | 1.00 |
| Depression before age 20 (child/teen onset) | 851 (67.6) | | 201 (56.8) | | 856 (50.4) | | Adverse effect | | 1.56 | | 1.32-1.84 | 1.09E-07 | 0.00 |
|  |  | |  | |  | | Positive effect | | 1.16 | | 0.91-1.49 | 2.41E-01 | 1.00 |
| **Sensitivity Analyses** |  | |  | |  | |  | |  | |  |  |  |
| PPD, restricted to women with no prior or child/teen onset depression | 172 (48.0) | | 63 (47.0) | | 292 (38.1) | | Adverse effect | | 1.77 | | 1.29-2.43 | 4.63E-04 | 0.01 |
|  |  | |  | |  | | Positive effect | | 1.99 | | 1.23-3.21 | 4.85E-03 | 0.08 |
| PMDD, restricted to women with no prior or child/teen onset depression | 11 (3.1) | | 12 (9) | | 8 (1) | | Adverse effect | | 2.93 | | 1.15-7.45 | 2.43E-01 | 1.00 |
|  |  | |  | |  | | Positive effect | | 5.6 | | 1.98-15.90 | 1.18E-03 | 0.02 |
| PPD, restricted to women with prior and child/teen onset depression | 215 (35) | | 40 (28.8) | | 200 (31.2) | | Adverse effect | | 1.26 | | 0.87-1.81 | 2.18E-01 | 1.00 |
|  |  | |  | |  | | Positive effect | | 1.49 | | 0.78-2.83 | 2.26E-01 | 1.00 |
| PMDD, restricted to women with prior and child/teen onset depression | 38 (6.2) | | 11 (7.9) | | 12 (1.9) | | Adverse effect | | 4.27 | | 1.68-10.8 | 2.24E-03 | 0.04 |
|  |  | |  | |  | | Positive effect | | 5.8 | | 1.8-18.7 | 3.22E-03 | 0.05 |
| **Association with polygenic score for major depression** |  | |  | |  | |  | |  | | | | |
|  |  | |  | |  | |  | | Relative risk ratio | | CI | P | adjusted P (Bonferroni) |
| Polygenic score (PGS) for MD (standardized using mean and SD of neutral OCP mood effect group) | Mean=0.15 | | Mean=0.07 | | 0 | | Adverse effect | | 1.18 per SD increase in PGS | | 1.09-1.27 | 3.57E-05 | 0.00 |
|  |  | |  | |  | | Positive effect | | 1.07 per SD increase in PGS | | 0.95-1.21 | 2.45E-01 | 1.00 |
| **Sensitivity Analysis** |  | |  | |  | |  | |  | |  |  |  |
| MD PGS (restricted to women with no prior or early onset depression and standardized using mean and SD of neutral OCP mood effect group within this restriction): negative mood effect | Mean=0.24 | | Mean=0.02 | | Mean=-0.00 | | Adverse effect | | 1.27 per SD increase in PGS | | 1.11-1.44 | 3.05E-04 | 0.00 |
|  |  | |  | |  | | Positive effect | | 1.00 per SD increase in PGS | | 0.83-1.21 | 9.76E-01 | 1.00 |

Table S3. Comparison across groups according to year of pill initiation - logistic regression results for those who experienced OCP adverse mood effect: Association of OCP adverse mood effect with depressive disorders.

|  | Initial use of OCP pre 1990 | | | | | | Initial use of OCP during or post 1990 | | | | | |
| --- | --- | --- | --- | --- | --- | --- | --- | --- | --- | --- | --- | --- |
|  | % Adverse mood effect group (n=417) | % No mood effect group (n=955) | Relative risk ratio | CI | P | Padj | % Adverse mood effect group (n=924) | % No mood effect group (n=870) | Relative risk ratio | CI | P | Padj |
| **Association with depression** |  |  |  |  |  |  |  |  |  |  |  |  |
| Peripartum depression (PPD) | 60.4 | 43.5 | 1.9 | 1.45-2.47 | 2.41E-06 | 0.00 | 31.4 | 25.1 | 1.38 | 0.97-1.98 | 7.66E-02 | 1.00 |
| Premenstrual dysphoric disorder (PMDD) | 5.5 | 1.8 | 3.13 | 1.59-6.15 | 9.24E-04 | 0.01 | 4.8 | 1 | 7.23 | 2.15-24.3 | 1.37E-03 | 0.02 |
| Perimenopausal depression | 5.8 | 9.3 | 0.62 | 0.32-1.2 | 1.54E-01 | 1.00 | 7.1 | 3.7 | 1.72 | 0.073-40.4 | 7.40E-01 | 1.00 |
| Depression before starting OCP (prior depression) | 41.2 | 30.5 | 1.63 | 1.26-2.10 | 1.63E-04 | 0.00 | 59.2 | 55.1 | 1.13 | 0.93-1.38 | 2.22E-01 | 1.00 |
| Depression before age 20 (child/teen onset) | 50.9 | 34.9 | 1.9 | 1.49-2.43 | 2.25E-07 | 0.00 | 75.1 | 67.2 | 1.34 | 1.07-1.68 | 1.17E-02 | 0.19 |
| **Sensitivity Analyses** |  |  |  |  |  |  |  |  |  |  |  |  |
| PPD, restricted to women with no prior or child/teen onset depression | 58.4 | 38.8 | 2.02 | 1.38-2.95 | 2.71E-04 | 0.00 | 37.8 | 36.4 | 1.28 | 0.68-2.40 | 4.46E-01 | 1.00 |
| PMDD, restricted to women with no prior or child/teen onset depression | 2.8 | 1.1 | 2.58 | 0.78-8.56 | 1.22E-01 | 1.00 | 3.3 | 0.9 | 4.05 | 0.798-20.5 | 9.15E-02 | 1.00 |
| PPD, restricted to women with prior and child/teen onset depression | 61.2 | 50.8 | 1.43 | 0.88-2.34 | 1.49E-01 | 1.00 | 26.8 | 19.3 | 0.99 | 0.55-1.79 | 9.71E-01 | 1.00 |
| PMDD, restricted to women with prior and child/teen onset depression | 6.1 | 2.9 | 2.21 | 0.80-6.10 | 1.27E-01 | 1.00 | 6.2 | 1.3 | 5.19 | 1.99-13.5 | 7.61E-04 | 0.01 |

Table S4. Comparison across groups according to year of pill initiation - logistic regression results for those who experienced OCP positive mood effect: Association of OCP positive mood effect with depressive disorders.

|  | Initial use of OCP pre 1990 | | | | | | Initial use of OCP during or post 1990 | | | | | |
| --- | --- | --- | --- | --- | --- | --- | --- | --- | --- | --- | --- | --- |
|  | % Positive mood effect group (n=161) | % No mood effect group (n=955) | Relative risk ratio | CI | P | Padj | % Positive mood effect group (n=217) | % No mood effect group (n=870) | Relative risk ratio | CI | P | Padj |
| **Association with depression** |  |  |  |  |  |  |  |  |  |  |  |  |
| Peripartum depression (PPD) | 54 | 43.5 | 1.76 | 1.18-2.61 | 5.06E-03 | 0.08 | 25.8 | 25.1 | 1.87 | 0.99-3.53 | 5.37E-02 | 0.86 |
| Premenstrual dysphoric disorder (PMDD) | 6.2 | 1.8 | 3.71 | 1.59-8.66 | 2.48E-03 | 0.04 | 8.8 | 1 | 13.7 | 3.53-52.8 | 1.51E-04 | 0.00 |
| Perimenopausal depression | 6.7 | 9.3 | 0.74 | 0.31-1.79 | 5.00E-01 | 1.00 | 0 | 3.7 | 0 | NA | 9.30E-01 | 1.00 |
| Depression before starting OCP (prior depression) | 28.6 | 30.5 | 0.92 | 0.62-1.36 | 6.83E-01 | 1.00 | 58.9 | 55.1 | 1.18 | 0.85-1.63 | 3.20E-01 | 1.00 |
| Depression before age 20 (child/teen onset) | 38.7 | 34.9 | 1.16 | 0.81-1.65 | 4.27E-01 | 1.00 | 70.1 | 67.2 | 1.2 | 0.84-1.71 | 3.23E-01 | 1.00 |
| **Sensitivity Analyses** |  |  |  |  |  |  |  |  |  |  |  |  |
| PPD, restricted to women with no prior or child/teen onset depression | 50 | 38.8 | 1.86 | 1.10-3.15 | 2.12E-02 | 0.34 | 41.3 | 36.4 | 2.52 | 0.75-8.49 | 1.35E-01 | 1.00 |
| PMDD, restricted to women with no prior or child/teen onset depression | 6.8 | 1.1 | 6.58 | 2.07-20.9 | 1.42E-03 | 0.02 | 13 | 0.9 | 16.9 | 3.30-86.9 | 6.97E-04 | 0.01 |
| PPD, restricted to women with prior and child/teen onset depression | 57.9 | 50.8 | 1.21 | 0.55-2.66 | 6.34E-01 | 1.00 | 17.8 | 19.3 | 2.11 | 0.64-6.93 | 2.19E-01 | 1.00 |
| PMDD, restricted to women with prior and child/teen onset depression | 7.9 | 2.9 | 2.9 | 0.71-11.8 | 1.37E-01 | 1.00 | 7.9 | 1.3 | 6.69 | 2.14-20.9 | 1.09E-03 | 0.02 |
